# Carina® and Esteem®: A Systematic Review of Fully Implantable Hearing Devices

**DOI:** 10.1371/journal.pone.0110636

**Published:** 2014-10-17

**Authors:** Janaina Oliveira Bentivi Pulcherio, Aline Gomes Bittencourt, Patrick Rademaker Burke, Rafael da Costa Monsanto, Rubens de Brito, Robinson Koji Tsuji, Ricardo Ferreira Bento

**Affiliations:** 1 Department of Otolaryngology, Hospital Central da Polícia Militar, Rio de Janeiro, Brazil; 2 Department of Otolaryngology, University of São Paulo School of Medicine, São Paulo, Brazil; 3 Department of Otolaryngology, Banco de Olhos de Sorocaba Hospital, São Paulo, Brazil; College of Medicine, Taiwan

## Abstract

**Objective:**

To review the outcomes of the fully implantable middle ear devices Carina and Esteem regarding the treatment of hearing loss.

**Data Sources:**

PubMed, Embase, Scielo, and Cochrane Library databases were searched.

**Study Selection:**

Abstracts of 77 citations were screened, and 43 articles were selected for full review. From those, 22 studies and two literature reviews in English directly demonstrating the results of Carina and Esteem were included.

**Data Extraction:**

There were a total of 244 patients ranging from 18 to 88 years. One hundred and 10 patients were implanted with Carina and with 134 Esteem. There were registered 92 males and 67 females. Five studies provided no information about patients’ age or gender. From the data available, the follow-up ranged from 2 to 29.4 months.

**Data Synthesis:**

The comparison of the results about word recognition is difficult as there was no standardization of measurement. The results were obtained from various sound intensities and different frequencies. The outcomes comparing to conventional HAs were conflicting. Nevertheless, all results comparing to unaided condition showed improvement and showed a subjective improvement of quality of life.

**Conclusion:**

There are still some problems to be solved, mainly related to device functioning and price. Due to the relatively few publications available and small sample sizes, we must be careful in extrapolating these results to a broader population. Additionally, none of all these studies represented level high levels of evidence (i.e. randomized controlled trials).

## Introduction

Hearing aids (HAs) are external listening devices that provide amplification and are traditionally used to treat hearing loss. Nevertheless, conventional HAs can lead to technical problems like feedback, requirement of regular maintenance, insufficient high-frequency gain for individuals with “ski-slope” hearing loss, inability to participate in water activities, sound distortion, effect of the ambient noise reaching the microphones and occlusion effect [1]–[5]. Clinical conditions like skin problems, malformation of the external ear and otitis can also limit their use [1], [2], [4], [5]. In addition, social factors (social stigma and cosmetic issues) may be mentioned [1], [3], [4].

The implantable devices are alternatives developed to promote greater comfort to patients with hearing loss bypassing the limitations of sound transmission through the external auditory meatus while keeping an external microphone, as it resides completely underneath the skin behind the ear.

The field of fully implantable middle ear devices (MEDs) is promising. Few studies are now available and they lack high level of evidence. This review aims to analyze the indications, the pre-operative assessment and mainly the effectiveness of the Carina system (Otologics LLC of Boulder, Colorado, USA) and the Esteem device (Envoy Medical Corporation, USA).

## Materials and Methods

A literature search was performed regarding the fully implantable hearing devices Carina and Esteem on July, 2014, using Pubmed, Embase, Scielo and Cochrane databases. The keywords used were “carina” AND “ear”, “esteem” AND “ear”, “fully implantable hearing aid”, “esteem” AND “Envoy”. Additional filters were used: English language, human subjects; the period of publication was set to 2000–2014. Duplicates were excluded at this point. The abstract of all the resulting studies were read, and after removing studies that did not comply with the inclusion/exclusion criteria, the remaining studies were read in full.

The criteria for study selection were as follows:

- Inclusion criteria:

–Case reports, prospective and retrospective studies referring to the outcomes of CARINA and ESTEEM implants.

- Exclusion criteria:

–Studies that did not review the results after the implantation of the device.

The results obtained in the different studies selected for appraisal were then gathered.

**Figure 1:**
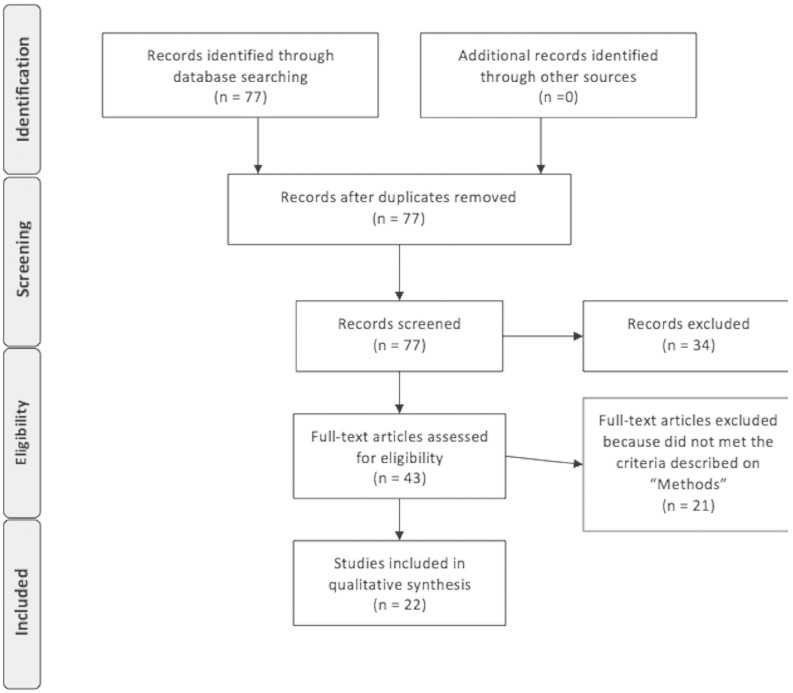
Decision process of the selection of the studies included in this review. Adapted from: Moher D, Liberati A, Tetzlaff J, Altman DG, The PRISMA Group (2009). Preferred Reporting Items for Systematic Reviews and Meta-Analyses: The PRISMA Statement. PLoS Med 6(6): e1000098. Doi:10.1371/journal.pmed1000097.

A flowchart of the decision process involved into the studies selection can be viewed below (Figure 1).

## Results

The search resulted on 77 citations. Titles and abstracts of 43 papers were screened as potentially relevant articles and selected for full review. Twenty-two original articles and two literature reviews met the study’s eligibility criteria and were included in this review.

There were a total of 244 patients ranging from 18 to 88 years. One hundred and 10 patients were implanted with Carina and 134 with Esteem. There were registered 92 males and 67 females. Five studies provided no information about patients’ age or gender. From the data available, the follow-up ranged from 2 to 29.4 months (Table 1).

**Table 1:** Demographic characteristics of the studies.

Study	Year	Patients(n)	MED	Gender	MeanAge	Side	MeanFollow-up(mths)
**Barbara et al.**	2009	6	Esteem	NR	NR	NR	NR
**Barbara et al.**	2011	18	Esteem	NR	NR	NR	NR
**Bruschini et al.**	2009 and 2010	8	Carina	7 M, 1F	46.6	6R, 2L	16.9
**Chen et al.**	2004	7	Esteem	2F, 5 M	64.4	4R, 3L	10
**Deveze et al.**	2010	1	Carina	1F	63	1R	6
**Gerard et al.**	2012	13	Esteem	9F, 4 M	NR	9R, 4L	4
**Jenkins et al.**	2007 and 2008	20	Carina	10F, 10 M	62.8	9R, 11L	2
**Kam et al.**	2012	6	Carina	3F, 3 M	48.5	4R, 2L	12
**Kraus et al.**	2011	57	Esteem	19F, 38 M	52.9	35R, 22L	12
**Lachowska et al.**	2012	1	Carina	1F	18	1R, 1L	9
**Lefebvre et al.**	2009	6	Carina	NR	NR	NR	12
**Martin et al.**	2009	11	Carina	7F, 4 M	50.8	5R, 6L	24
**Memari et al.**	2011	10	Esteem	7 M, 3F	32.7	6L, 4R	29.4
**Monini et al.**	2013	15	Esteem	8F, 7 M	NR	NR	NR
**Murali**	2009	3	Esteem	2 M, 1F	28,6	2R, 1L	NR
**Shohet**	2011	5	Esteem	NR	NR	NR	12
**Siegert**	2007	5	Carina	4 M, 1F	31,4	NR	3
**Tringali et al.**	2008	1	Carina	1 M	14	1R	2
**Tringali et al.**	2009	1	Carina	1F	48	1R	15
**Zenner and Rodriguez**	2010	50	Carina	NR	NR	NR	NR

n – number; MED – middle ear device; mths – months; M – male; F – female; NR – not reported.

### 1. History

The use of MEDs for treatment of hearing loss became part of the clinical practice in Europe and United States. The Vibrant Soundbridge (Med-El Corporation, Innsbruck, Austria), which has an external auditory processor, was the first device routinely used in the last 90′s [6], [7]. The first fully implantable MEDs was developed in 1999 by Implex GmbH from Germany, but was withdrawn from the market because of technical and economic problems [3].

Two fully implantable MEDs are currently available for use: the Esteem used in Europe and in the United States and the fourth generation of the Carina, approved for use in all European Community countries and on phase II efficacy studies for the Food and Drug Administration (FDA) approval.

### 2. Mechanisms

The mechanism of MEDs is correct hearing loss by stimulating the ossicular chain or the round window directly [6]–[10]. The Esteem is the first totally implanted MEDs based on piezoelectric technology. It consists of a titanium dual-channel sound processor housed in a temporal bone niche, a nonrechargeable battery and 2 piezoelectric transducers (a “sensor” and a “driver”). The “sensor” is placed on the body of the incus where it can detect tympanic membrane vibration, converts it to electrical signal and sends it to the sound processor. The sound processor, on the other hand, amplifiers, filters, and sends the stimulus to the piezoelectric transducers (the “driver”) that modifies the electrical sign back to mechanical energy and causes vibration to the stapes. Therefore, the device is microphone free and the sound is received directly by the eardrum/ossicular chain [4], [11], [12].

Piezoelectric crystals are more efficient than electromagnets, once the power consumption is reduced because there is no need to create a magnetic field^1^. Consequently the lithium battery life is now compatible with total implantability [1]. The expected battery life is 4.5 years with continuous use (24 hours per day/7 days per week) to 9 years (if only used for 8 hours per day) [3], [11]–[14]. The battery changing may be performed as a surgical procedure under local anesthesia [1], [13].

To prevent feedback phenomenon from the device, implantation requires separation of the incustapedial joint and resection of a segment (about 2 millimeters) of the long process of the incus [1], [14]. Some studies have demonstrated that the Esteem device may provide effective bandwidth output that exceeds 8 kHz [15]. Low distortion permits increasing acoustic gain (can reach up to +55 dB) without compromising audibility [11], [14]. An open ear canal eliminates the occlusion effect [4]. Natural directionality and auricular filtering (at high frequencies) are preserved [13].

The Carina system is the successor of the semi-implantable MET (*Middle Ear Transducer*) system [3], [8]. It consists on a microphone, battery, magnet, digital signal processor, transducer and a connector. Sounds are captured by the microphone and relayed to the sound processor within the implant capsule. The sound processor analyses the sound information, amplifies it according to the programmed settings, and converts it into electrical signals that are relayed to the transducer attached to the incus. This transducer translates electrical signals into a mechanic motion that directly stimulates the ossicular chain or the round window. Ossicles are not disarticulated [3], [9], [13], [16]–[18].

The battery is charged by a coil placed on the skin over the implant, using a belt or waistband. It may be performed daily during 1 to 1.5 hours and each charge lasts 32 hours [3], [10], [16], [17]. As stated by the manufacturer, the battery lifetime is at least 10 years, after which the entire electronic capsule must be surgically removed for replacement. The middle ear transducer is not removed.

### 3. Implantation technique

#### 3.1. Carina system

The usual Carina system implantation is performed through a post-auricular incision with a posterior small atticotomy (about 2 cm wide) [18] to expose the body of the incus and the head of the malleus. The arm of the mounting bracket of the device can be modified to place the device on the incus and is fitted to the mastoid cortex using bone screws. Bone beds for the device and the microphone must be drilled so that the electronics capsule and the microphone can be positioned and secured [9], [19].

There are 3 convenient microphone placement locations: anterior and superior to the external auditory canal (temporalis region), posterior to the external auditory canal (retro-auricular region), and on the mastoid tip. It is noteworthy that the microphone is very sensitive to changes in the tissue thickness over time, resulting in feedback. Thus, it may be placed in a region of minimal tissue thickening changes during head and neck movements, which is not the case of the mastoid tip. It is also necessary to avoid the contraction effects of the sternocleidomastoid muscle [19]. Moreover, there is no consensus regarding the optimal placement of the microphone [9].

The tip of the transducer is advanced into the hole on the incus and the positioning is evaluated using software specifically developed by Otologics (Transducer Loading Assistant) to ensure correct placement of the device [17].

The tip of Carinás transducer can be crimped to different tips such as stapes head, stapes superstructure, stapes footplate or round window [9]. The round window may be used in patients for whom multiple ossiculoplasty procedures have been unsuccessful and particularly when the stapes footplate is fixed or no longer accessible [9], [17]. This transducer tip can also be extended by applying a small titanium ball and placed on the body of the incus [9].

The implantation of Carina to stimulate the round window is performed through a post-auricular incision with a posterior tympanotomy to expose the round window niche. The round window membrane movement is checked by the mobilization of the stapes or the long process of the incus. The transducer is placed on the round window with or without placement of the incus. The tip of transducer is adapted by clipping modified total ossicular replacement prosthesis (TORP) to the end of the transducer or can be put in contact with the staples footplate or even coupled to a stapedotomy piston. The other end of the TORP is placed on a fascia graft protecting the round window membrane. This surgical technique allows the implantation of device despite of a nonfunctioning ossicular chain and an abnormal middle ear anatomy as long as round window membrane is present to receive the tip of the transducer [6], [9].

#### 3.2. Esteem Device

The ear with the poorest functional hearing is selected for implantation. If both ears are equal in performance, the candidate can choose the side to be implanted. The procedure is performed under general anesthesia [1].

A post-auricular incision is made and a bone recess is fashioned posterior to the mastoid to house the sound processor. A tympanomastoidectomy is performed widely exposing the facial recess to accommodate the driver. The chorda tympani nerve is sacrified in about 60% of the cases [13.14]. The intact ossicular motion can be measured using a laser doppler vibrometer. The incus and stapes are disarticulated and the distal 1 to 3 mm of the long process of the incus is gently removed using either malleus nipper or a cutting laser to prevent a mechanic feedback. Transducers are contained in the mastoid cavity with hydroxyapatite cement so their piezoelectric crystals are positioned. The sensor is interfaced with the incus using glass ionomeric cement and the driver is cemented to the stapes [1], [13].

In a study to detect the site of maximum ossicular motion that would be optimal for attachment of the sensor portion of the protesis, Chung et al. [2] used a laser doppler vibrometer to measure the vibrational responses at 7 locations on the middle ear ossicles. They observed that maximum vibrational motion of the middle ear is deliverable to the piezoelectric transducer of Esteem through the superior part of the malleus head, on the lateral part of the incus body, and on the superior part of the incus body near the incudomalleal joint.

After implant placement, the entire system is tested and postoperative functional gain is estimated. If gain is deemed inadequate, the implant is repositioned to improve performance.

Both devices, Carina and Esteem are turned on in about 6 to 8 weeks after implantation [6], . There is an implant programming called “commander” that is used by audiologist for follow-up to program each patient’s device. The patient himself can also modify the filtering of background noise levels, adjust volume, and place the device on stand-by mode using a remote control component called “personal programmer” [1], [22], [23].

### 4. Indications

The fully implantable hearing devices are indicated mainly as an alternative treatment for moderate to severe hearing loss in patients with normal and abnormal middle ears who either do not benefit from conventional HAs or choose not to wear them [1], [4], [5], [10], [13]. Patients with low tegmen mastoideum or tympani, anteriorly displaced sigmoid sinus, small facial recess, or laterally displaced facial nerve are not candidates [13], [14], [19], [20].

Recently, Carinás indications has been extended to patients with ossicular defects in whom conventional ossiculoplasty itself would not restore sufficient hearing function. The Carina device can be deployed in several places permitting the contact with the ossicular chain. Round window implantation is also a possibility and bypasses the normal conductive pathway to the cochlea and apart from the condition of ossicular chain or external ears such as congenital auricular atresia [7], [8], [9], [22].

### 5. Outcomes

The advantages of fully implantable HAs led to greater patient satisfaction. The good performance of them is due to several factors as absence of occlusion effect or feedback, cosmetic advantage, and the possibility of use the device every day [11]. These factors were considered although some of the performance outcome measures with the Carina were lower than those pre-operatively with HAs in the study of Bruschini et al. [17].

Listening to body sounds (muscle movement, heartbeat, breathing, hair noise, local stimulating noise) was not a complaint of 13 patients implanted with Esteem studied by Gerard et al. [14] but was related by the patient bilaterally implanted with Carina by Lachowska et al. [16] and was cited by Martin et al. [9].

The Hearing in Noise Test (HINT) is a more reliable test for the evaluation of daily conditions. The ability to understand in quiet was 88% and in noise was 62% for the 7 patients implanted with Esteem studied by Chen et al. [1] Shohet et al. [15] reported improvement in 4 of 5 patients over HAs. None of these two studies showed statistical significance. The HINT for the 57 patients (also implanted with Esteem) studied by Kraus et al. [13] was not worse than in quit conditions. For the Carina device, Jenkins et al. [19] reported a deterioration of HINT after 6 months of implantation but had dramatically improved after refitting and remained better than the patients’ own hearing aid until 1-year follow up.

The Esteem Questionnaire was designed by St. Croix Medical to evaluate subjective questions specifically about the Esteem device. Kraus et al. [13] used this tool to assess the quality of life of 57 patients at 12 months. The majority of subjects considered their device to be equal to or much better than their own HA in all subcales (clarity of sound, speech in noise, natural voices, understanding conversation, self-confidence and active lifestyle).

The Client oriented scale improvement (COSI) questionnaire is used to document a patient’s goals/needs and to measure improvements in hearing ability [4], [11]. Before the application of this test, every patient selects 5 major listening situations that he/she would like to improve and assign a maximum score of 5 for each situation. Monini et al. [4] applied this test to the 2 groups of their study 3 months after implantation. In the goup A (moderate to severe SNHL) the mean COSI final score was 13.5 for conventional HA and 22.7 for the Esteem (p = 0.00001). In the group B (severe to profound SNHL) there was no statistical significance (p = 0.270) for the benefit. The mean COSI scores in the study of Barbara et al. [11] changed from 17.7 (in itinere) to 20.6 (final score) for moderate SNHL and from 18.1 (in itinere) to 18.2 (final score) for severe SNHL.

The Glasgow Benefit Inventory (GBI) is an 18-item questionnaire, with scores ranging from −100 to +100, developed especially to measure patients’ benefit after otorhinolaringological interventions [11]. This test was used in Barbara et al. [11] study and showed only a slightly better score in the moderate hearing loss population.

#### 5.1. Fully implantable devices compared with hearing aids

Chen et al. [1] reported that the functional gain for the Esteem implanted in was similar to HAs at 0.5, 1, 2 and 3 KHz and this gain decreased at 3 kHz. Monini et al. [4] found that a mean gain difference of 13 dB favorable to the Esteem device, compared to HAs in both groups moderate-to-severe and severe-to-profound sensorineural hearing loss (SNHL), but with no statistical significance. Otherwise, Memari et al. [20] (n = 10) showed that average gain in 5 frequencies ranged from no gain in 1 patient to 20 dB in another 1. Barbara et al. [11] referred improvement from 70 dB to 48 dB in the whole sample. Shohet et al. [15] reported a functional gain of 22 dB with this same device.

In the study by Lefebvre et al. [6], the 6 patients who underwent implantation of Carina transducing sound via the round window showed essentially the same thresholds for frequencies above 3 kHz comparing to conventional HAs.

The average functional gain at 500, 1 k and 2 k Hz was found to be 35.6 dB and 35.0 dB in 6 patients with Carina and the conventional HA, respectively, revealing an insignificant difference according to Kam et al. [22] Gain on frequencies above 3 kHz is generally limited but residual hearing at such frequencies can still be maintained or slightly improved [14].

Zenner and Rodriguez [24] (n = 50) reported that the average functional gain varied by frequency between 25 and 30 dB for audiometric test frequencies of 5 and 6 KHz.

The word recognition (WR) improvement for Esteem comparing to conventional HA’s varied among the studies. While Chen et al. [1] found an index of improvement with Esteem of 17% compared to HAs, Kraus et al. [13] found that 62% of their subjects (n = 52) had improvement, 27% were the same, and 11% were worse. Neither studies presented information of statistical significance. Monini et al. [4], showed that WR raised to 55% with conventional HA and to 66% with the Esteem in the group with moderate-to-severe SNHL. In the group with severe-to-profound SNHL the improvement was to 46% with conventional HA and to 57% with the Esteem. However the difference was not statistically significant. Carina results in WR were conflicting in all articles studied. Zenner and Rodriguez [24] (n = 50) reported that a significant improvement, up to 82% correct, in speech discrimination scores was obtained. The case report of Deveze et al. [26] showed improvement from 40% to 80% with Carina device at 65 dB, comparing to the patient’s own HAs. Nevertheless, Kam et al. [22] found insignificant difference between conventional HAs and the Carina in terms of WR both in quiet and in noise.

The APHAB (Abbreviated Profile of Hearing Aid Benefit) scale was applied before and after implantation in some studies [1], [10], [11], [13], [18], [19], and it consists of questionnaires in four areas: EC (Ease of Communication), BN (Background Noise), RV (Reverberation) and AV (Aversiveness) [1], [10], [19]. According to *Instruction for Manual Scoring of the APHAB*, a significant benefit has occurred if a difference of 22% is obtained for the EC, RV, or BN score [1]. Jenkins et al. [19] found that patients preferred the Carina over their own HAs for all questionnaires. According to Kam et al. [22], the APHAB scale for Carina and HA were 84.9 and 37.2, respectively.

#### 5.2. Fully implantable devices compared with unaided

Comparing the outcomes of Carina implantation to unaided patients, the mean functional gain was 29 dB and 24 dB (p  = 0.0004) in Martin et al. [9] and Bruschini et al. [17] respectively. Tringali et al. [7] showed a mean improvement of 39 dB with no information about statistical significance. Bruschini et al. [18] reported a mean functional gain of 26.4 dB (p = 0.0000001).

Five patients suffering from congenital auricular atresia submitted to Carina had functional gain of 36 dBHL in 4 frequencies by pure tone audiometry (1, 2, 3 e 4 kHz) as shown by Siegert et al. [8].

Kraus et al. [13] (n = 57) studied the 12-month results of the Esteem and found that the functional gain was 27 dB for 48 patients and 4 of them were stable at ±10 dB. Gerard et al. [14] found that the mean gain was 25±11 dB and the best results were obtained at frequencies between 500 and 3 k Hz.

According to Gerard et al. [14], the Esteem device, when compared to unaidaded, showed a mean WR gain of 64±33% at 50 dB SPL. At 50 dB, the WR improved from 10% at unaided condition and 23% with HA to 78% with Esteem according to Shohet et al. [15] A great improvement was reported by Barbara et al. [11] (42% to 79% in a group with moderate hearing loss and 30% to 72% in a group with severe hearing loss). Murali et al. [22] showed that postoperative WR on an average for all their 3 patients for closed set and open set were 100% and 95% respectively. All Carina results in WR comparing to unaided condition showed improvement. WR mean melioration according to the studies of Bruschini et al.[17], [18] were 18% to 58% [17] and from 32.5% to 68.75% [18].

When the APHAB scale was applied, Martin et al. [9] showed significant benefit for Carina over unaided conditions for EC (from 49.8 to 19.9%) and RV (from 57.7 to 44.8%). AV increased (25.8 to 38.6%). Chen et al. [1] found that the average score for AV was −33 and −6 for HAs and Esteem compared to unaided condition, respectively, what means a 27% improvement of Esteem over the HAs. On this same comparison, Kraus et al. [13] showed that the mean difference on the global scale was 8.9±2.6 (p<0.01). The APHAB questionnaire revealed 85% of satisfaction improvement with Esteem compared to HAs in the 4 subscales in the study of Gerard et al. [14] Their 2 dissatisfied patients underwent revision surgery for poor functional results.

#### 5.3. Fully implantable devices activated and inactivated

The middle and inner ear conditions were evaluated by some authors. Tringali et al. [21], Martin et al. [9] and Bruschini et al. [18] found no significant changes postoperatively, indicating minimal surgical trauma during Carina implantation. Jenkins et al. [19] observed no pre- nor post implantation differences for bone conduction and slight differences in pre- and post implant for air conduction.

As Esteem implantation induces an additional conductive hearing loss, Monini et al. [4] observed a conductive threshold shift of 35 dB on average over the whole frequency range. Barbara et al. [11], [25] showed a bone conduction threshold worsening from baseline after Esteem implantation. For Kraus et al. [13] the average change was mean –0.8±1.1 dB. At 12 months only one patient had a threshold shift from 55 to 75 dB at 4 kHz. In the other hand, Chen et al. [1] and Gerard et al. [14] showed no significant changes of cochlear function by comparing bone conduction threshold before and after implantation of Esteem.

### 6. Complications and adverse events

Occasional feedback for the Carina device was cited by Kam et al. [22] and Bruschini et al. [18], but it was resolved through the fine-tuning of the fitting and gain reduction (Table 2). Bruschini et al. [18] reported a case of a patient who had the microphone implanted in the tip of mastoid and complained of too much feedback noise, especially when turning the head. It was necessary to reposition the implant.

**Table 2:** Complications related to the fully implantable middle ear devices.

Study	Year	MED	Patients(n)	AdverseEffects (n)	SurgicalComplications(n)	DeviceAdverseEffects (n)	Revisions(n)
**Bruschini** **et al.**	2009	Carina	5	NR	None	Feedback (5)	1
**Bruschini** **et al.**	2010	Carina	8	Extrusion of the cable of microphone (1),Psychologicalproblems andexplantation (1)	NR	Feedback (1),Device failure (1)	2
**Chen** **et al.**	2004	Esteem	7	Woundcomplication (1)	Temporary swelling ofthe lower eyelid, sorejaw, nausea, diarrhea,elbow pain, arm andhand pain, andnumbness (2)	No benefit (3),Low gain (3)	3
**Gerard** **et al.**	2012	Esteem	13	Secondaryhealing difficulty(1), recurrenttissue edema (1),S aureus woundinfection (2)	Temporary partialfacial palsy (1),rupture of chordatympani nerve (8),	Poor anddeterioratingfunctionalresults (4)	4
**Jenkins**	2007 and 208	Carina	20	fullness orpressure sensation(2), conductivehearing loss (4),lightheadedness(1), tinnitus (1),partial deviceextrusion (3), andmiddle eareffusion (3)		partial deviceextrusion (3),inability tocharge orestablishcommunication(2),increasedcharging timesbeyond 1.5 hours	
**Kam** **et al.**	2012	6	None	None	feedback		1
**Kraus** **et al.**	2011	Esteem	57	Taste disturbance(24), middle eareffusion (18),pain (8), dizziness(9), tinnitus (7),headache (3),infection (2)	Facialparesis/paralysis (4)	Limitedbenefit (4)	3
**Martin** **et al.**	2009	Carina	11	Infection (2),vertigo (1),tinnitus (1)	NR	Hearing loss(1), poor soundtransmission (1)	1
**Memari** **et al.**	2011	Esteem	10	NR	Temporary facialweakness (1)	Nobenefit (2)	NR
**Murali** **et al.**	2009	Esteem	3	None	transient facialparesis (1)	NR	0

n – number; MED – middle ear device; NR – not reported.

Martin et al. [9] reported 2 cases of postoperative infection after Carina implantation and the need of reoperation in both. Another patient from their study had a decide failure but the patient declined revision surgery.

Jenkins et al. [19] (n = 20) cited fullness or pressure sensation in 10% of the subjects using Carina, middle ear effusion and partial device extrusion in 15%, vertigo and tinnitus in 5% and conductive hearing loss in 20%. Three of the 20 have not been reached until 1-year follow-up of and 16 patients have asked to be explanted and reimplanted with a device modification. Lefebvre et al. [6], on the other hand, showed no complication up to 12-month follow-up of Carina.

For 57 patients with Esteem, Kraus et al. [13] reported 133 adverse events in 52 patients. There were 5.2% of revision surgeries, 3.5% developed wound infection (one them required explantation), 5.2% evolved with facial paresis (1 patient maintained House-Brackmann level II). Still about Esteem, Chen et al.^1^ reported temporary swelling of the lower eyelid, sore jaw, nausea, diarrhea, elbow pain, arm and hand pain, and numbness. A device-related wound complication occurred that ultimately required implant removal in one subject.

The minor complications reported by Gerard et al. [14] with the Esteem device were: temporary partial facial palsy (7.6%), disruption of chorda tympani nerve (61.5%), revision surgery because of healing difficulty (7.6%) and 23% of revision surgeries for poor functional results. The major complication was the implant removal because of wound infection (15.3%).

## Discussion

This systematic review evaluated the outcomes of Carina and Esteem implantation, devices currently available for use. The fully implantable MED is an alternative for many patients with limited benefits using conventional HAs, even those with only cosmetic issues. The indications are now not only for SNHL. Some authors had shown great outcomes using these devices for patients with external ear and ossicular chain defects, extending the indication for conductive and mixed hearing losses.

All the studies showed improvement of sound field threshold from unaided to aided conditions with fully implantable MED. About gain, there are conflicting results among the different studies. Some of them have no statistical significance. Some studies reported a functional gain but with a limited benefit on frequencies above 3 kHz [1], [6], [14].

The concern about middle ear conditions and cochlear function after implantation of some authors lies in the issues of surgical procedure. No changes in bone conduction before and after implantation were observed in most of the studies for the Carina. As Esteem implantation induces an additional conductive hearing loss, 2 studies showed a conductive threshold [4], [11].

The comparison of the results about word recognition is difficult to make because there was no standardization of measurement. The results were obtained from various sound intensities and different frequencies. Also, some studies reported only the improvement; some showed the pre- and post-operative results; some offered only the graphics. All results comparing to unaided condition showed improvement. The results comparing to conventional HAs were conflicting.

For APHAB scale, all studies that evaluated the comparison between unaided and aided conditions and between the middle-ear device and conventional HAs for both Esteem and Carina showed benefit [1]. It means a subjective improvement of quality of life.

The complications involved in Carina and Esteem implantation were also studied. The main complications related to Esteem implantation were related to the surgical procedure. It should be kept in mind that the need for explantation will demand reconstruction of the ossicular chain. Otherwise, the hearing threshold will increase due to the overlapping of conductive hearing loss on a preexisting SNHL.

For Carina device, despite of the events related to surgical procedure, many studies showed device malfunction or failure with a need for revision surgery or explantations. This fact may be due to charging issues.

## Conclusion

The use of fully implantable MED is now part of otology practice all around the world and this field is promising for those dissatisfied with their current conventional air-conduction hearing aids. Although there are still some problems yet to be solved mainly related to device functioning and price.

Due to the relatively few publications available and small sample sizes, we must be careful in extrapolating these results to a broader population. Additionally, none of all these studies represented level high levels of evidence (i.e. randomized controlled trials).

## Supporting Information

Checklist S1 **PRISMA Checklist.**
(DOC)
